# Acoustic Streaming-Based 3D Cell Focusing and Plasma Separation

**DOI:** 10.3390/mi17050560

**Published:** 2026-04-30

**Authors:** Jingjing Zheng, Qian Wu, Zhenheng Lin, Xuejia Hu, Liqing Qiao, Genliang Li, Jinkun Luo

**Affiliations:** 1 College of Artificial Intelligence, Putian Electronic Information Industry Technology Research Institute, Putian University, Putian 351100, China; lintom2023@ptu.edu.cn; 2 Department of Electronic Engineering, School of Electronic Science and Engineering, Xiamen University, Xiamen 361102, China; wuqian_s@163.com (Q.W.); xjhu@xmu.edu.cn (X.H.); 3 College of Intelligent Manufacturing, Putian University, Putian 351100, China; qiaolq@ptu.edu.cn; 4 College of Computer and Data Science, Putian University, Putian 351100, China; humrry@foxmail.com (G.L.); jkluolucky@ptu.edu.cn (J.L.)

**Keywords:** acoustofluidics, cell focusing, plasma separation, integrated microfluidic

## Abstract

Separating plasma from small-volume blood samples is important for rapid blood analysis in point-of-care testing. Microfluidic approaches provide flexible platforms for plasma extraction, but many methods either require complex pretreatment or rely on sheath-assisted or multi-step operations. In this study, we present an acoustofluidic platform that enables sheath-free three-dimensional (3D) focusing of blood cells and downstream plasma extraction in an integrated microchip. The device employs symmetric cavity-trapped bubbles to generate acoustic streaming under acoustic excitation, thereby reconstructing the local flow field and driving suspended cells toward a stable central region of the channel. Based on this mechanism, blood cells are concentrated toward the middle outlet, while plasma is collected from the two side outlets. The device remains operable over a range of inflow conditions through acoustic-voltage adjustment. Using diluted simulated blood samples, the platform achieved a plasma recovery of approximately 71% and a plasma purity of approximately 99%. In addition, cell-viability tests indicated good biocompatibility under the tested operating conditions. Owing to its simple structure, integrated design, and sheath-free operation, this platform shows potential for future miniaturized sample-preparation applications. However, further validation using real whole blood and clinically relevant plasma-quality metrics will be required in future studies.

## 1. Introduction

Plasma is an essential clinical sample for disease diagnosis because it contains diverse biomarkers, including proteins and exosomes. Plasma is rich in multiple biomarkers, and its efficient separation is fundamental for accurate disease diagnosis and therapeutic monitoring [1,2,3]. High-speed centrifugation is currently considered the gold standard for plasma separation due to its high processing efficiency. However, this method depends on bulky and costly equipment and typically requires large sample volumes (~mL), which restricts its use in real-time processing, particularly for micro-volume samples. In addition, high-speed centrifugation can induce mechanical damage to blood cells [4,5]. Although membrane filtration provides a more economical and efficient alternative for plasma extraction, certain cellulose membranes are susceptible to pore clogging by blood cells, which reduces filtration efficiency, prolongs processing time, and may even cause red blood cell rupture [6,7,8,9,10].

Microfluidic technology enables precise manipulation of fluids and cells within microscale channels, offering exceptional flexibility and control [11]. To date, various lab-on-a-chip strategies have been developed for plasma separation, which can be broadly classified into passive and active methods. Passive approaches rely on microchannel design or intrinsic fluid dynamics to achieve plasma separation and have attracted considerable interest due to their simplicity and low cost [12,13,14,15,16,17]. However, their performance strongly depends on factors such as channel geometry, fluid properties, sample volume, and flow rate, which limit their separation efficiency when handling small-volume blood samples. In contrast, active methods enhance operational precision and control by applying external physical fields, such as magnetic, electric, or acoustic fields—thereby enabling the extraction of higher-purity plasma components [18,19,20,21,22,23,24]. However, many active approaches—such as magnetic separation requiring bead labeling or dielectrophoresis requiring high-frequency signals—depend on complex external equipment and specific labeling or pretreatment steps. These requirements not only increase system complexity but may also compromise the native activity and stability of biological components. Among various active methods, acoustic flow control technology exhibits distinct advantages [25,26]. Its non-contact, label-free, and highly biocompatible nature provides an ideal platform for cell manipulation and blood component separation, with broad applications in biomedical fields such as cell capture, alignment, and separation [27,28,29,30,31,32,33,34,35,36]. While acoustofluidics has emerged as a powerful tool for cell manipulation, the specific mechanisms employed for blood separation vary widely in their underlying physics and engineering trade-offs. Bulk acoustic wave (BAW) devices typically utilize half-wavelength resonances within microchannels to drive cells toward pressure nodes via primary acoustic radiation forces, achieving high-throughput focusing in architectures such as glass–silicon resonant cavities or disposable polymer chips [37]. Alternatively, surface acoustic wave (SAW) devices generate traveling or standing waves on piezoelectric substrates, offering precise two-dimensional manipulation with high frequencies but often requiring cleanroom-intensive fabrication of interdigitated transducers [38]. More recently, acoustofluidic approaches have been extended to include media gradients for density-based separation [39], photoacoustic effects for localized streaming, and diffractive acoustic fields for complex patterning [40,41,42]. Despite these advances, many of these methods rely on precise resonant alignment, high-frequency signal sources, or complex multi-step lithography, which can limit their portability and ease of integration.

In contrast to high-frequency bulk and surface acoustic wave techniques that often rely on precise resonant alignment or complex lithography, cavity-induced acoustic streaming at low frequencies provides a robust, sheathless focusing mechanism that operates effectively in simple single-layer PDMS channels, offering a compelling alternative for portable plasma separation applications. The use of oscillating bubbles trapped in lateral cavities has been established as a versatile mechanism for generating localized microstreaming, with foundational work demonstrating that Lateral Cavity Acoustic Transducers can deflect cells and particles into bifurcating channels via vortex-like streaming and subsequently achieve simultaneous on-chip pumping and size-based separation, while further studies have shown that oscillating microbubbles can selectively trap particles based on size and density. However, the translation of this bubble-based streaming mechanism to a dedicated, high-purity plasma separation platform—with quantitative recovery metrics and fully integrated portable electronics—has remained underexplored, and a critical practical challenge, namely the long-term stability of the gas–liquid interface during continuous flow, has not been systematically addressed in prior work. Herein, we do not claim the first use of bubble-induced acoustic streaming. Rather, the novelty of this work lies in the integration of symmetric dual-bubble vortices with a three-outlet microfluidic architecture to achieve sheath-free 3D blood cell focusing and continuous plasma extraction within a compact, voltage-tunable acoustofluidic platform. This approach circumvents the need for expensive cleanroom-fabricated interdigital transducers (IDTs) and complex sheath-flow regulation, offering a practical alternative for low-cost plasma separation.

This paper presents a microfluidic chip that leverages the above-described acoustic streaming mechanism to achieve rapid and continuous plasma separation via three-dimensional cell focusing. Coupled with a triple-outlet design, the system enables direct collection of high-purity plasma from both side outlets, yielding an experimentally measured plasma purity exceeding 99% and a recovery rate of approximately 71% in diluted simulated blood samples. Importantly, the present study further demonstrates that a deliberately retracted bubble interface within the symmetric dual-cavity configuration sustains a stable linear oscillation regime, thereby enabling reliable operation in a handheld, wirelessly controlled device, highlighting significant potential for point-of-care biomedical analysis.

## 2. Materials and Methods

### 2.1. Device Fabrication

The integrated platform consists of an acoustoelectronic system and an acoustic vortex microchip. The acoustoelectronic system comprises four main components: a power supply module, a signal generation module, a secondary amplification unit, and a Bluetooth-enabled control module. All modules are integrated onto a single processing board. The circuitry is tailored to specific requirements, enabling wireless operation of the piezoelectric ceramic (PZT). The acoustic vortex chip is composed of a PZT and a microfluidic chip. The microfluidic chip is fabricated using soft photolithography. In detail, the microfluidic chip is designed using AutoCAD 2020 software, which is then printed out as a plastic photomask for the subsequent UV exposure step. Silicon wafer is spin-coated with negative photoresist (SU8 2050, MicroChem, Newton, MA, USA) and exposed to UV light with the plastic photomask. Following baking and developing, a master mold with a thickness of 120 µm microfluidic channel is produced. PDMS is poured onto the master mold after eliminating air bubbles, which is then baked at 75 °C for 2 h to form a PDMS chip with a microchannel structure. This chip features one inlet and three outlets, with a main channel width of 200 µm, an intermediate outlet width of 60 µm, and side-outlet channels measuring 80 μm in width. Symmetric cavities, each 70 µm wide, are located on both sides of the main channel to generate symmetric acoustic vortices. Using a sine wave, a PZT element (20 mm), and an epoxy coupling method, the resonance frequency is approximately 26 kHz. After perforation and cleaning with alcohol, the PDMS is plasma-treated (PDC-002, HARRICK PLASMA, Shenzhen, China) for 2 min. The treated PDMS chip is then bonded to a glass substrate that is attached to the PZT. To precisely define the bubble interface, a liquid–gas displacement protocol was employed: the channels were first filled with DI water, followed by air injection to evacuate the main channel while trapping a small liquid volume at the cavity apex by surface tension. Finally, the acoustofluidic chip and the electronic circuits are integrated into a device with a drawer structure. The PDMS-glass chips can be cleaned, dried, and reused for multiple experimental runs without noticeable degradation in performance.

### 2.2. Sample Preparation

The MCF-7 breast cancer cell line is purchased from Wuhan Purcell Life Science Co. (Wuhan, China). Cells are cultured in Dulbecco’s modified Eagle’s medium (DMEM, Gibco, Waltham, MA, USA) supplemented with 10% fetal bovine serum (Gibco, USA) and 1% penicillin/streptomycin (Gibco, USA) at a CO_2_ incubator (37 °C and 5% CO_2_). Prior to the experiment, cells are detached by trypsin (Gibco, USA), collected and transferred to centrifuge tubes (BS-50-M, Biosharp, Hefei, China) for use. Blood samples are simulated using hematology control and purchased from Mindray Biomedical Electronics Co., Ltd. (Shenzhen, China). Blood-related experiments in this study were performed using diluted simulated blood prepared from a commercial hematology control. Specifically, 20 μL of the simulated blood sample was mixed with 380 μL of physiological saline before use. We note that the present study was therefore limited to diluted simulated blood rather than real whole blood.

### 2.3. Vitality Testing and Staining

In the biocompatibility experiments, MCF-7 cells are divided into two groups: an experimental group that undergoes acoustic vortex processing and a blank control group that receives no treatment. Both groups of cells are stained with Calcein-AM and PI solution (Sigma-Aldrich, St. Louis, MO, USA) and then observed under a fluorescence microscope. The survival rates of the cells in both groups are calculated and compared. Here, live cells are defined as green-fluoresce positive and red-fluoresce negative. For the CCK8 assay, both groups start with the same initial cell concentration and are maintained in an incubator. After incubation for 12 h, 24 h, and 36 h, the number of viable cells in each group is first counted to assess their growth capacity. Then, cell samples are co-cultured with CCK-8 solution (5 mg/mL) for 4 h. Finally, a microplate reader (Emax Precision, Irvine, CA, USA) is used to measure the absorbance at 450 nm for each sample, with five parallel samples analyzed per group to facilitate a comparison of results between the two groups.

### 2.4. Experimental Procedure

A diluted simulated blood sample is injected into the microchannel using an infusion pump. An acoustic signal is generated via a Bluetooth connection to a smartphone. The symmetrical cavities generated symmetrical acoustic vortices, directing blood cells to exit through the central outlet while plasma is collected from the side-outlet channels. To monitor plasma separation efficiency during the experiment, the device is mounted on an optical fluorescence microscope platform (Ti ECLIPSE, Nikon, Tokyo, Japan) and connected to a CMOS camera (ORCA Flash 4.0 v2, Hamamatsu, Japan). To ensure statistical independence, each data point represents a measurement from a freshly loaded microfluidic chip or a distinct cell culture well. For flow experiments, chips were thoroughly cleaned with ethanol and deionized water, dried, and re-primed between independent runs. For cell culture experiments, separate wells were seeded from the same parent culture but maintained and assayed independently.

### 2.5. Statistical Analysis

Quantitative data are presented as mean ± standard deviation unless otherwise specified in the figure captions. The number of replicates for each experiment is indicated in the corresponding figure caption or text where available. Statistical analysis for the proliferation experiment was performed as described in the manuscript using linear fitting of the time-dependent OD450 curves, followed by comparison of fitted slopes. Because some experiments in this proof-of-concept study were primarily intended to demonstrate device feasibility, the statistical depth is limited and should be expanded in future work.

## 3. Results and Discussion

### 3.1. The Design and Operating Mechanism of the Acoustic Vortex-Based Platform

This acoustofluidic cell sorting system utilizes the acoustic streaming effect to efficiently separate blood cells from plasma via acoustic wave-induced bubble oscillations. Upon activation of the acoustic signal, bubbles within symmetric cavities oscillate and induce a significant acoustic streaming effect, which alters the flow field in the microfluidic channel and generates gradient flow velocity in a specific region at the bubble boundary [43,44,45]. which can be approximated as follows:

(1) $$U_{0}\sim d\omega$$

Here, ‘d’ denotes the amplitude of the interface oscillation, and ‘ω’ signifies the angular frequency of the acoustic field. The first characteristic velocity of cavity-induced microflows, ‘$U_{0}$’ defines the flow field’s velocity. The thickness of the oscillatory boundary layer, ‘δ’, is expressed as:

(2) $$\delta \;\sim {\left(v/\omega \right)}^{1/2}$$

Here, ‘v’ represents the dynamic viscosity of the fluid. Within the oscillatory boundary layer, due to the equilibrium between viscous forces at the interface and nonlinear inertial forces (i.e., Reynolds stresses), the first-order periodic flow triggers a stable second-order flow. The magnitude of the stable microflow within the oscillatory boundary layer is given by [46,47]:

(3) $$U_{S}\;\sim U_{0}^{2}/\omega R$$

where ‘$U_{S}$’ denotes the flow velocity near the gas/liquid interface and defines the second characteristic velocity of cavity-induced microflows. ‘R’ represents the length scale of the oscillatory interface and can be defined as the equivalent radius of the gas/liquid interface. The above expressions are included to provide physical justification for the generation of steady acoustic streaming near the oscillating bubble interfaces, rather than to establish a full quantitative force decomposition for the blood cell trajectories in the present device.

**Figure 1:**
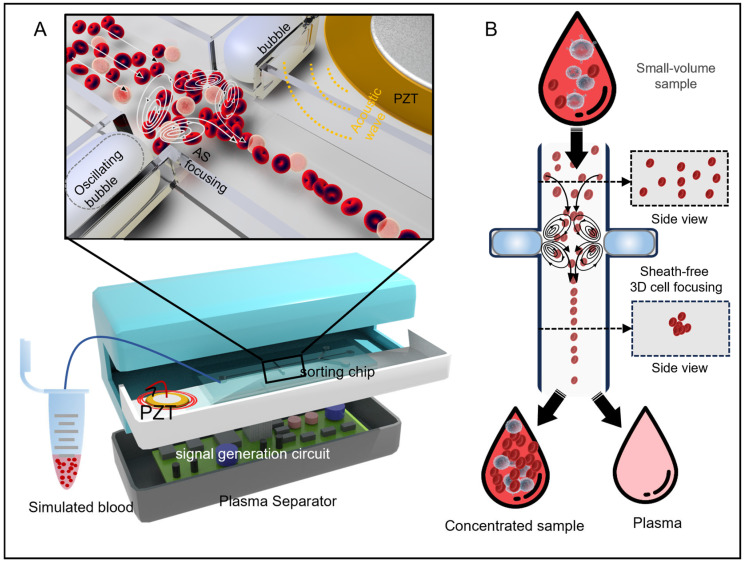
The integrated acoustofluidic device enables sheath-free 3D cell focusing and high-efficiency separation of blood cells and plasma. (**A**) Schematic of the acoustic vortex chip for 3D cell focusing. The microfluidic chip leverages a symmetric bubble structure to generate symmetric vortices under acoustic excitation, facilitating 3D blood cell focusing. (**B**) Schematic of the portable plasma separation device and the 3D cell focusing in the microfluidic chip.

As shown in Figure 1A, a micro-volume simulated blood sample is injected into the microfluidic channel and flows toward the oscillating gas–liquid interface. Blood cells are initially entrained by viscous drag within the flow field and are subsequently transported by the acoustic streaming along the streamlines. After undergoing a resonance cavity-like rotation, the cells reach a maximum deflection angle. The rapid decrease in fluid velocity from the channel wall to the center creates a balanced region at the 3D center of the channel, where the cells are ejected. Unlike bulk acoustic wave devices, in which the primary acoustic radiation force acts directly on particles, the focusing observed here is a consequence of cavity-induced acoustic streaming. The oscillating bubble generates a steady second-order flow (Equations (1)–(3)) that convects the surrounding fluid and drags the cells toward the low-velocity region at the channel center. The primary acoustic radiation force is negligible in this configuration due to the small acoustic contrast factor between the cells and the surrounding fluid, as well as the localized nature of the streaming field. The combination of the acoustic streaming effect and fluid drag force enables the acoustic vortex chip to achieve 3D focusing of the blood cells. When integrated with a microfluidic channel containing multiple outlets, this method efficiently separates blood cells from plasma.

Based on these acoustic vortex chips, a portable plasma separation device is designed as illustrated in Figure 1B. To minimize the size of the device, the acoustic signal generator, amplifier, and feedback measurement components are integrated into a single processing board (Figure S1A,B). The detailed scheme and architecture of this acoustic–electronic system are presented in Figure S1C,D. This system employs a signal amplification circuit module to amplify the signal power for driving the PZT transducer. The initial and output signals are measured (Figure S1E), demonstrating the system’s effective amplification capabilities. Further, the circuit calibration module is utilized to precisely control the output signals. Figure S1F indicates that the user-set signal fits well with the outputs, which can achieve a maximum of 40 VPP amplitudes without obvious distortion and deviation.

### 3.2. Characterization of the Acoustic Vortex Chips

**Figure 2:**
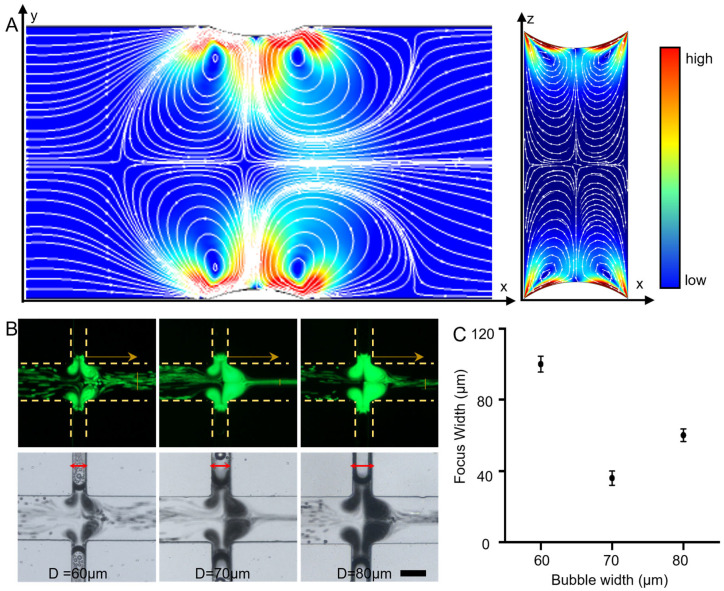
Simulation and experimental results of sound-induced symmetric acoustic vortices. (**A**) The left image shows the simulated top-view results of particle focusing, while the right image presents the corresponding side-view results. (**B**) Fluorescence and bright-field images of particle focusing effects under different bubble widths. Scale bar: 100 μm. Arrows indicate the direction of fluid flow. (**C**) The corresponding particle focusing width values for different bubble widths. Data are presented as mean ± standard deviation (SD). n = 5.

To evaluate the feasibility of the acoustic fluidic device, numerical simulations were performed using the finite element method, with particular emphasis on the acoustic streaming-induced focusing effect generated by symmetric bubbles. The simulations were based on a thermoviscous acoustics module, which accurately accounts for acoustic attenuation arising from viscous dissipation and thermal conduction. The model couples and solves the linearized Navier–Stokes, continuity, and energy equations, enabling analysis of the thin viscous and thermal boundary layers formed near the solid–liquid interface. Finally, the solver configuration (first-order time-domain solution, second-order steady-state solution) is presented. Figure 2A presents the simulated flow fields and streamlines in both the XY and XZ planes. The results reveal pronounced streamline convergence in the central region of the channel, corresponding to a low-pressure zone where the flow is relatively stagnant. This flow configuration enables effective focusing of particles and cells into this region. Because acoustic streaming is generated by oscillating bubbles within symmetric cavities, its efficiency is strongly influenced by bubble size. Therefore, we investigated the effect of bubble diameter (D = 60 μm, 70 μm, and 80 μm) on the focusing performance (Figure 2B). Under constant acoustic excitation (14.1 Vpp) and a fixed flow rate (200 µL/h), fluorescent polystyrene microparticles were introduced into devices with different bubble diameters. The measured focusing widths under each condition are summarized in Figure 2C. The results show that the focusing widths were approximately 100 μm for D = 60 μm, 36 μm for D = 70 μm, and 60 μm for D = 80 μm. Overall, the most concentrated and stable particle focusing occurred with a bubble diameter of 70 μm, indicating that this dimension provides optimal acoustofluidic focusing performance. Consequently, a bubble diameter of 70 μm was selected as the optimal parameter for subsequent experiments. Continuous microscopic observation confirmed that the gas–liquid interface remained stable with positional drift less than 5 μm over the duration of a standard experimental run, likely due to the dynamic equilibrium of dissolved gas under low-frequency oscillation. It should be noted that the simulation employs a simplified geometric configuration in which the bubble interface is flush with the main channel wall, in order to qualitatively demonstrate the symmetric vortex structure. The experimental setup, by contrast, employs a slightly recessed bubble interface (approximately 5–10 μm from the main channel sidewall), which helps improve interface stability during continuous operation. Although this recessed geometry reduces the absolute flow velocity within the main channel, the centrally symmetric vortex topology and the resulting three-dimensional convergence capability are still preserved. Because of this geometric simplification, the numerical results presented here are intended as qualitative support for the focusing mechanism rather than as a strict quantitative predictor of the experimental interface configuration.

### 3.3. Parameter Optimization of Acoustic Vortex-Based Focusing

To evaluate the focusing effect, the focusing width (FW) is defined as the distance 230 μm from the lower edge of the bubble and is used in subsequent experiments. The primary parameters that can influence the focusing effect are the acoustic intensity exerted on the PZT transducer and the inflow rate of particle injection. As shown in Figure S2, increasing the driving voltage enhances the bubble oscillation amplitude and strengthens the associated acoustic streaming, thereby promoting tighter focusing and a reduced FW. In contrast, increasing the sample flow rate strengthens the axial through-flow and tends to broaden the focused stream, resulting in a larger FW. Thus, effective focusing requires a balance between streaming-induced confinement and pressure-driven transport.

**Figure 3:**
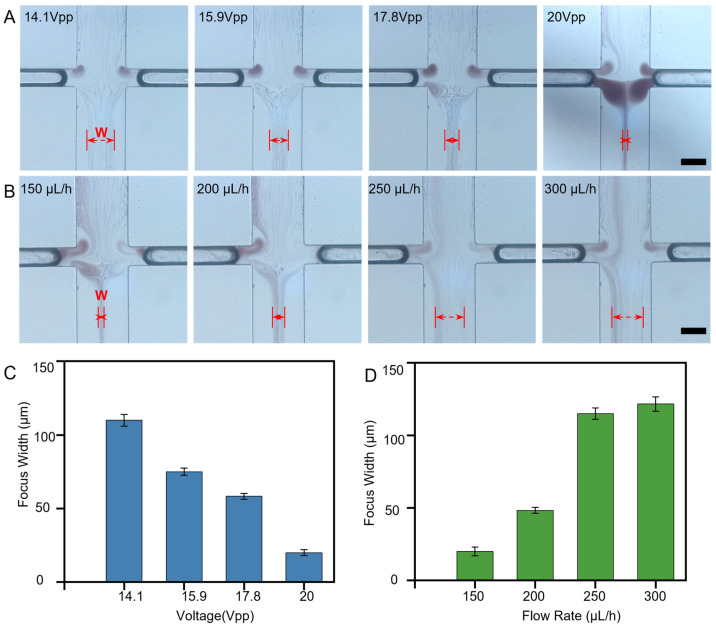
Optimization of acoustic focusing parameters. (**A**) The focusing width decreases with the increase in acoustic voltage. Scale bar: 100 μm. (**B**) Under the same acoustic voltage, the focusing width increases with the increase in flow rate. Scale bar: 100 μm. (**C**) Variation in the focusing width of blood cells with acoustic voltage. Data are presented as mean ± standard deviation (SD). n = 5. (**D**) Variation in the focusing width of blood cells with flow rate under the same acoustic voltage. Data are presented as mean ± standard deviation (SD). n = 5.

The simulated blood sample is used for testing, using the same acoustic vortex chip and parameters. Results show that the FW progressively decreases with increasing acoustic intensity (Movie S1). Next, the flow rate is increased at a fixed acoustic intensity, and the variation in FW is recorded, revealing that it expanded with increasing flow rate (Movie S2). No measurable degradation in focusing performance was observed during continuous operation over the duration of a standard experimental run, as evidenced by the stable focusing streams shown in Movies S1 and S2. Blood cell focusing images acquired by the microscope are shown in Figure 3A,B. Quantitative analysis is shown in Figure 3C,D, specifically, when acoustic-voltage increase to 20 Vpp, the FW can decrease to as small as 20 µm. Operation at such high amplitudes induces low-frequency oscillations in the shape of the bubble interface, likely due to the system operating outside the linear region, resulting in nonlinear, non-spherical oscillation modes. These shape instabilities caused intermittent perturbations in the focusing stream, reducing the reliability of continuous plasma collection. In contrast, at 14.1 Vpp, the bubble operates within a stable linear oscillation regime, producing a reliable focusing width of 36 ± 3 μm with consistent purity and minimal run-to-run variability. Therefore, 14.1 Vpp was selected as the optimal operating point for stable, continuous-flow separation, prioritizing reproducibility and robustness over the absolute minimum focusing width.

### 3.4. Biocompatibility of Acoustic Vortex Chips

**Figure 4:**
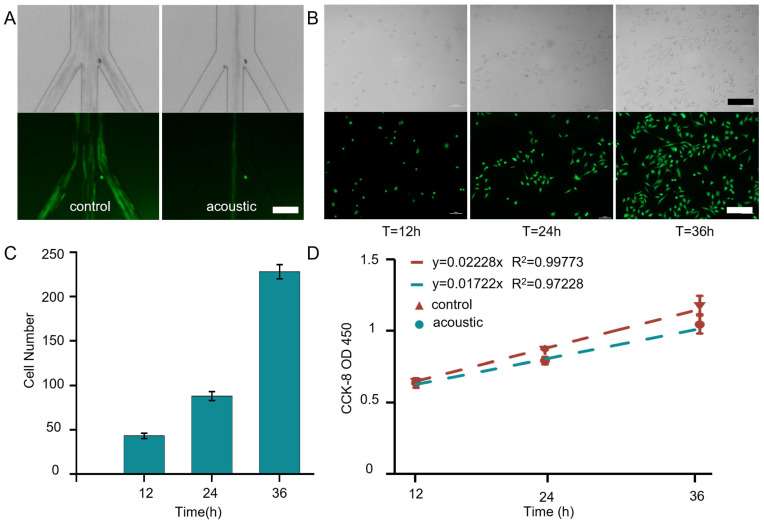
(**A**) Bright-field and fluorescence images of cells separated when an acoustic signal is applied. Cells stained with Calcein-AM exhibit green fluorescence. Scale bar: 130 µm. (**B**) Bright-field and fluorescence images of collected cells proliferating over time during several days of culture. Scale bar: 130 µm. (**C**) Cell numbers at different times during proliferation tests. Data are presented as mean ± SD. n = 5 independent culture wells per time point per group. (**D**) OD450 values of acoustically treated and untreated cells measured with CCK-8. Time-dependent OD450 curves are linearly fitted using the mean and variance from four independent groups. Data are presented as mean ± SD. n = 5 independent culture wells per time point per group.

Biocompatibility is another significant property of the acoustic vortex chip used for cell separation and analysis. Figure 4A illustrates the validation using MCF-7 breast cancer cells stained with Calcein-AM. MCF-7 breast cancer cells were selected for viability and proliferation assays because adherent epithelial cell lines are generally more sensitive to shear stress and acoustic perturbation than suspended blood cells. Demonstrating that the acoustic vortex does not compromise the viability or proliferative capacity of these sensitive cells provides a conservative, worst-case validation of the platform’s biocompatibility. MCF-7 cells are divided into two groups: a control group with no treatment and an acoustic group where cells are focused using the acoustic vortex. Cells collected from the central outlet of both groups are stained with Calcein-AM and PI for viability analysis. Figure S3A shows the immunofluorescent results of cells with and without acoustic treatment, while Figure S3B presents a quantitative comparison of cell viability between the two groups. The analysis reveals that the cell viability of the control and acoustic groups is 99.4% and 97.6%, respectively, confirming the biocompatibility of this system. Furthermore, cells in the acoustic group are cultured for 12, 24, and 36 h, and their proliferative growth over time is monitored. Figure 4B displays bright-field and fluorescence images of MCF-7 cells, where cells are stained with Calcein-AM. The quantitative data on cell numbers are illustrated in Figure 4C. Over time, the cell population exhibits significant growth, indicating an intact proliferative capacity. Figure 4D presents the results of the CCK-8 assay. Linear regression analysis was performed on the curves, where the slope represents the proliferative capacity.

Comparison of the fitted slopes using an F-test revealed no statistically significant difference (*p* > 0.05). The high cell viability observed here is consistent with previous reports demonstrating that low-intensity acoustic fields and mild acoustic streaming do not compromise cell membrane integrity or induce significant apoptosis. Acoustic-based manipulation techniques are generally regarded as biocompatible due to the non-contact nature of the forces and the low energy deposition into the fluid medium [48,49]. However, it is important to note that the present biocompatibility assessment is confined to short-term viability and proliferation assays performed immediately after acoustic exposure. Long-term functional studies, such as cytokine release profiles or hematopoietic differentiation assays, fall outside the scope of this work. Therefore, while the 97.6% viability confirms that the acoustic streaming field is non-destructive under the operating conditions used here, further investigations would be required to fully validate the clinical suitability of the extracted plasma and the separated blood cells.

### 3.5. Plasma Extraction

**Figure 5:**
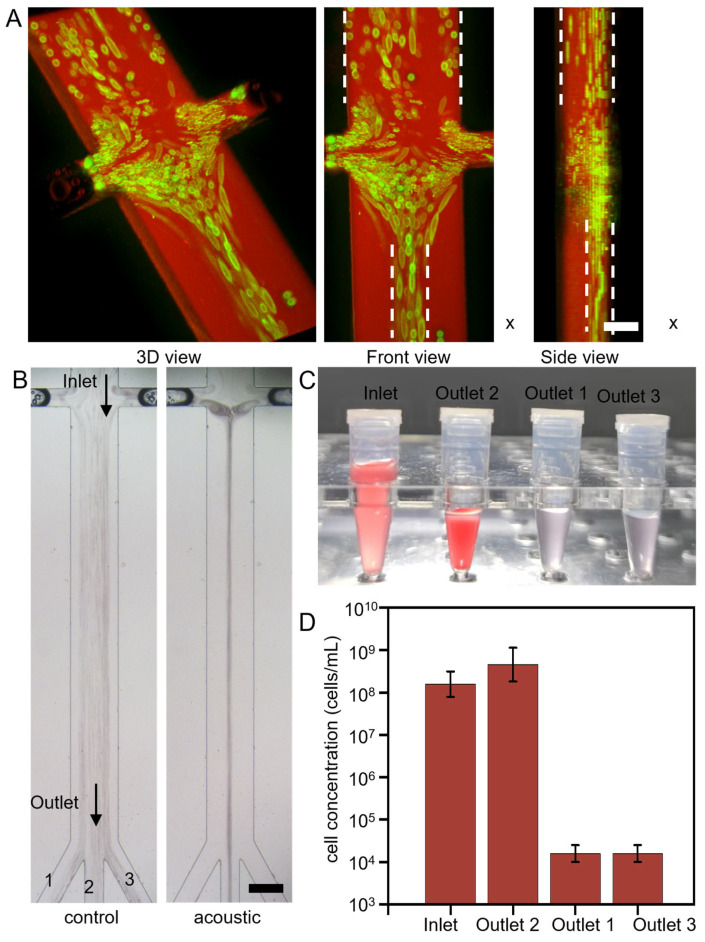
Sorting of simulated blood samples via acoustic streaming. (**A**) Confocal microscopy images of green fluorescent PS microspheres demonstrating three-dimensional focusing, where the solution is stained with rhodamine B (red) to mark the channel boundaries. The white dashed line indicates the width variation before and after focusing. The right panel (XZ plane) illustrates the vertical confinement of the particle stream; note that due to the limited height of the side channels, particles are focused toward the upper region of the channel rather than the exact mid-height. Scale bar: 60 µm. (**B**) Trajectories of blood cells in the chip with and without the application of acoustic signals. Scale bar: 240 µm. (**C**) Photographs of the initial diluted blood sample and collected samples from outlets 1/3 (plasma) and outlet 2 (blood cells). (**D**) Quantitative assessment of blood cell concentration post-acoustic separation. Data are presented as mean ± SD. n = 5 independent separation experiments. The confocal images qualitatively illustrate 3D focusing behavior; statistical position-distribution analysis was not included in the present study.

Figure 5A illustrates the three-dimensional focusing performance of the acoustic vortex chip as observed using confocal microscopy. Fluorescently labeled polystyrene (PS) microspheres, which emit green fluorescence under laser excitation, clearly delineate the particle focusing trajectory. The channel architecture is visualized using Rhodamine B, which emits red fluorescence when excited by a 560 nm laser, thereby outlining the microfluidic conduit. The left panel shows the overall 3D focusing effect; the middle panel displays the focused particle stream in the XY plane, with white dashed lines indicating the channel width and the central focusing trajectory; and the right panel presents the XZ plane, where white dashed lines mark the channel height, revealing that the focused particles are primarily confined to the upper region. Due to the limited height of the lateral channel, the particles are focused at the top of the channel rather than in the middle. It should be noted that lateral (XY) focusing has been quantitatively characterized via focusing width measurements under varying conditions (Figure 2C and Figure 3C,D). The confocal images presented here serve as a qualitative visualization of the three-dimensional flow behavior rather than a complete statistical proof of robust 3D centering. Quantification of the full three-dimensional position distribution via particle tracking velocimetry remains an important direction for future work. Furthermore, Figure 5B demonstrates the device’s performance in blood processing across the entire XY plane. The central outlet is used to collect concentrated cellular components, whereas the two side outlets are employed to extract purified plasma. This process is further demonstrated dynamically in Movie S3. As shown in Figure 5C, samples were collected from three independent outlets. The results indicate that the majority of blood cells were collected through the central outlet, whereas high-purity plasma was obtained from the two side outlets. To quantify the separation performance, blood cell concentrations at the inlet and outlets were measured using a hemocytometer under optical microscopy. Plasma Recovery Rate ($R_{p}$): Defined as the ratio of the plasma volume collected from the two side outlets to the total plasma volume in the outlet, whereas high-purity plasma was obtained from the two side outlets. To quantify the separation performance, blood cell concentrations at the inlet and outlets were measured using a hemocytometer under optical microscopy. Plasma Recovery Rate ($R_{p}$): Defined as the ratio of the plasma volume collected from the two side outlets to the total plasma volume in the inlet sample. $Rp=\frac{Q_{side}}{Q_{inlet}}$, where *Q* denotes volumetric flow rate. Plasma Purity ($P_{p}$): Defined as the fraction of the collected side-outlet sample that is cell-free plasma, $Pp=1-\frac{Blood\;cell\;count\;in\;side\;outlets}{Total\;volume\;of\;side\;outlet\;sample}$. Cell Depletion Efficiency ($E_{d}$): Defined as the fraction of inlet blood cells removed from the side outlets. $E_{d}=1-\frac{C_{cell,side}}{C_{cell,inlet}}$. Enrichment Factor (*EF*): Defined as the ratio of blood cell concentration in the central outlet to that in the inlet, *EF=*$\frac{C_{cell,center}}{C_{cell,inlet}}$. As shown in Figure 5D, the device achieved effective plasma separation, with a plasma recovery rate of approximately 70% and a plasma purity of approximately 99%.

The key performance metrics of this platform were compared with those of representative passive and active microfluidic plasma separation systems reported in the literature (Table S1). Passive methods (such as hydrodynamic filtration or sedimentation) typically offer high-throughput (100–1000 µL/h), but often require sheath flow or complex channel geometries to achieve comparable purity. Bulk acoustic wave (BAW) and surface acoustic wave (SAW) devices offer excellent biocompatibility and high plasma recovery rates; however, they typically require precise alignment of piezoelectric transducers or the fabrication of expensive finger-shaped transducers (IDTs), thereby increasing device complexity and operating costs. In contrast, this device utilizes a symmetric double-bubble flow mechanism to achieve approximately 99% plasma purity and about 71% recovery at a flow rate of 200 µL/h. Notably, the device requires no sheath flow, relies solely on a low-cost PZT transducer, and is manufactured using standard soft lithography processes. Although its throughput is moderate compared to certain high-flow-rate passive systems, the combination of adjustable acoustic focusing, a simple manufacturing process, and low equipment costs makes this method particularly attractive for point-of-care testing and resource-limited settings where external pumps and complex optical systems are unavailable. These results demonstrate that acoustic vortex chips are capable of achieving efficient three-dimensional cell focusing and separation. While the present work validates the separation of the cellular fraction from the plasma background using simulated blood, we acknowledge that a full clinical assessment requires further analysis. Future studies will focus on validating the platform with whole human blood to quantify hemolysis levels, assess platelet carryover, and evaluate protein recovery and biomarker compatibility.

## 4. Conclusions

In summary, we developed a portable acoustofluidic platform for plasma extraction based on bubble-induced acoustic streaming and sheath-free three-dimensional blood cell focusing. Symmetric cavity-trapped bubbles generated localized streaming vortices that reconstructed the channel flow field and concentrated blood cells toward the center outlet, enabling plasma-rich fractions to be collected from the side outlets. Using diluted simulated blood, the device achieved a plasma recovery of approximately 70% and a plasma purity approaching 99%. The platform integrates microfluidic separation with compact electronic actuation and demonstrates the feasibility of bubble-streaming-based plasma extraction in a miniaturized format.

At the same time, the present study has several limitations. The experiments were performed using diluted simulated blood rather than real whole blood; quantitative lateral and vertical position distributions were not measured; long-term bubble stability, device lifetime, and chip-to-chip variation were not systematically evaluated; and clinically relevant plasma-quality metrics such as hemolysis, platelet carryover, and analyte preservation were not assessed. Therefore, the current work should be regarded as a proof-of-concept study, and further validation will be required before translational application in disease diagnosis or biomarker analysis.

## Data Availability

The original contributions presented in this study are included in the article/Supplementary Materials. Further inquiries can be directed to the corresponding author.

## Supplementary Materials

The following supporting information can be downloaded at: https://www.mdpi.com/article/10.3390/mi17050560/s1.
