# Supplementary material for: Acoustic Streaming-Based 3D Cell Focusing and Plasma Separation
Source: Micromachines (Basel). 2026 Apr 30;17(5):560. doi: 10.3390/mi17050560 (PMC13208755; doi:10.3390/mi17050560)
Supplement: Supplementary file 1 [file micromachines-17-00560-s001.zip › micromachines-4257672-supplementary.pdf]

# Supporting Information

## Acoustic streaming-based 3D cell focusing and Plasma Separation

Jingjing Zheng,[1]* Qian Wu,[2] Zhenheng Lin,[1] Xuejia Hu,[2] Liqing Qiao[3] Genliang Li[4] and Jinkun Luo[4]

[1] College of Artificial Intelligence, Putian Electronic Information Industry Technology Research Institute，Putian University, Putian, Fujian 351100, China;
[2] Department of Electronic Engineering, School of Electronic Science and Engineering, Xiamen University, Xiamen 361102, China
[3] College of Intelligent Manufacturing, Putian University, Putian 351100, China
[4] College of Computer and Data Science, Putian University, Putian 351100, China
Jingjing Zheng and [1]Qian Wu contributed equally.

**Corresponding Author**

Jingjing Zheng - College of Artificial Intelligence, Putian University, Putian, 351100, China; Putian Electronic Information Industry Technology Research Institute, Putian University, Putian, Fujian 351100, China; Email: zhengjj@ptu.edu.cn

**S1. Diagram of the integrated plasma separator unit.** 3
**S2. Characterization plots of PS fluorescent microspheres simulating blood cell parameters.** 4
**S3. Blood separation and cell activity before and after acoustic signaling.** 5
**S4. Comparison of this acoustic fluidic device with other microfluidic plasma separation systems.** 6
**S5. Supplementary movies** 7

**S1. Diagram of the integrated plasma separator unit.**

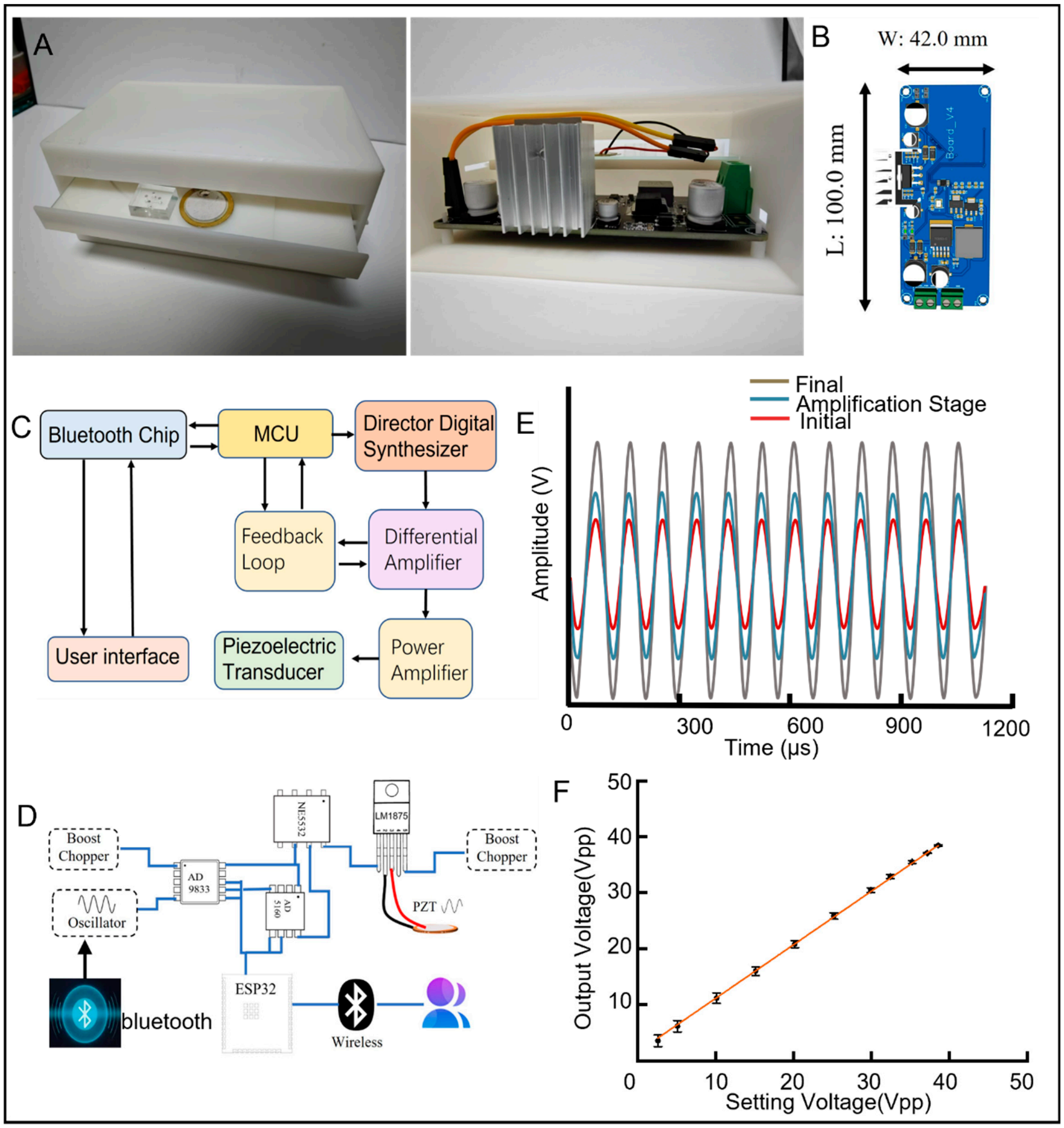


**Figure S1** (A) Photograph of the integrated device, including its overall structure and internal components. (B) Dimensions of the internal processing board. (C) and (D) Workflow diagrams illustrating the synergy between internal modules. (E) Waveforms of the initial, first-stage, and second-stage amplified output signals. (F) Relationship between the set voltage and the device output voltage.

**S2. Characterization plots of PS fluorescent microspheres simulating blood cell parameters.**

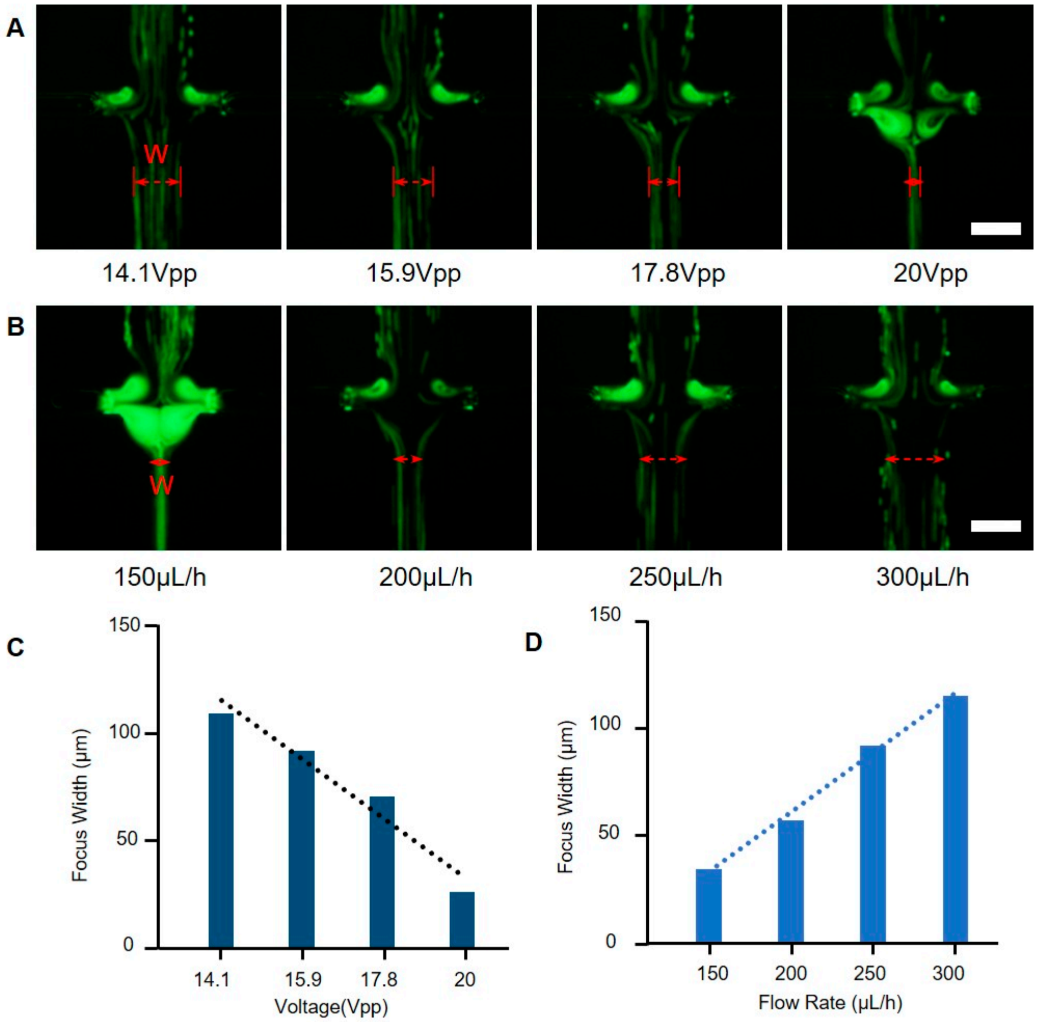


**Figure S2** Characterization chart of PS fluorescent microspheres simulating blood cell parameters. (A) Focusing effect of different acoustic intensities at the same flow rate. Scale bar:120μm. (B) Focusing effect at different flow rates under the same acoustic intensity. Scale bar:120μm. (C) Focusing width under different acoustic intensities at the same flow rate. (D) Focusing width at different flow rates at the same acoustic intensity.

**S3. Blood separation and cell activity before and after acoustic signaling.**

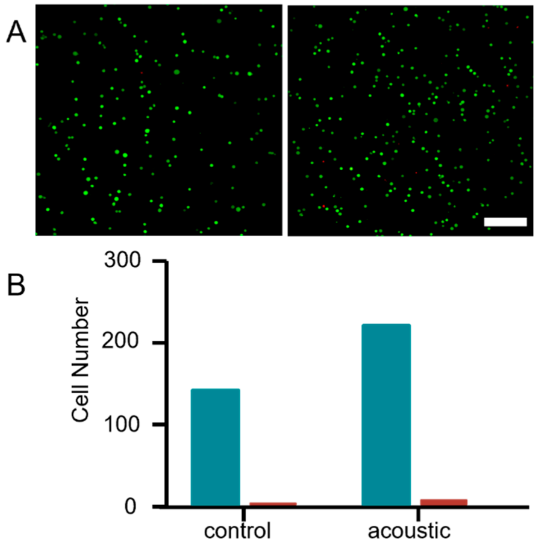


**Figure S3** (A) Fluorescence images of the distribution of living dead cells before and after the action of acoustic signals. Scale bar:340μm. (B) Number of living dead cells before and after the acoustic signal action.

## S4. Comparison of this acoustic fluidic device with other microfluidic plasma separation systems.

**Table S1** Comparison of the present acoustofluidic device with representative passive and active microfluidic plasma separation systems.

| Feature | This Work | Passive Methods | BAW | SAW | Other Acoustic |
|---|---|---|---|---|---|
| **Separation Mechanism** | Symmetric dual-bubble acoustic streaming | Hydrodynamic / Filtration | Primary acoustic radiation force (pressure node) | Surface acoustic wave radiation force | Photoacoustic / Diffractive acoustic fields |
| **Sample Type** | Diluted simulated blood | Whole / Diluted blood | Whole / Diluted blood | Whole / Diluted blood | Model particles / Diluted blood |
| **Throughput (μL/h)** | 200 | 100–1000 | 100–600 | 10–300 | < 100 |
| **Plasma Purity** | ~99% | 90–98% | 85–95% | 90–98% | > 95% (limited data) |
| **Plasma Recovery** | ~71% | 60–80% | 70–90% | 60–85% | Not reported |
| **Sheath Flow Required** | No | Yes/No | Usually Yes | Usually Yes | Design-dependent |
| **Fabrication Complexity** | Low (standard soft lithography) | Low–Medium | Medium (alignment needed) | High (IDT fabrication) | High (laser / metamaterial) |
| **Equipment Cost** | Low (single PZT) | Low | Medium | High | Very High |
| **Biocompatibility Validated** | Yes (cell viability 97.6%) | Not typically | Yes | Yes | Limited |
| **Key References** | This work | [12-17] | [18,24] | [27,28] | [37-42] |

**S5. Supplementary movies**

**Movie S1 (separate file).** Changes in the focusing effect of blood cells with increasing applied acoustic intensity.

**Movie S2 (separate file).** Changes in the focusing effect of blood cells with increasing applied injection flow rate.

**Movie S3 (separate file).** Dynamics of blood cells at the exit as the acoustic signal opens with increasing acoustic intensity outflow.
